# Supplementary material for: Medical students’ crisis-induced stress and the association with social support
Source: PLoS One. 2022 Dec 1;17(12):e0278577. doi: 10.1371/journal.pone.0278577 (PMC9714810; doi:10.1371/journal.pone.0278577)
Supplement: S1 Table — Note: Questions and scoring emotional-informational support in English, with in italic-grey the Dutch version used for the data-collection. Minimum score: 0, maximum score: 16. Alpha reliability cohort 2018 = 0.895. Alpha reliability cohort 2019 = 0.907. (PDF) [file pone.0278577.s001.pdf]

S1 Table. Measurement emotional-informational support.

How often are the following forms of support available to you when you need it? *Hoe vaak zijn de volgende vormen van steun voor jou beschikbaar als je het nodig hebt?*	**None of the time** *Nooit*	**A little of the time** *Weinig*	**Some of the time** *Soms*	**Most of the time** *Meestal*	**All of the time** *Altijd*
Someone you can count on to listen to you when you need to talk *Iemand op wie je kunt rekenen en die naar je luistert als je wilt praten*	0	1	2	3	4
Someone whose advice you really want *Iemand wiens advies je graag wilt*	0	1	2	3	4
Someone to share your most private worries and fears with *Iemand waarmee je persoonlijke zorgen en angsten kunt delen*	0	1	2	3	4
Someone to turn to for suggestions about how to deal with a personal problem *Iemand die je suggesties kan geven over hoe je met een persoonlijk probleem om moet gaan*	0	1	2	3	4

Note: Questions and scoring emotional-informational support in English, with in italic-grey the Dutch version used for the data-collection. Minimum score: 0, maximum score: 16.

Alpha reliability cohort 2018 = 0.895

Alpha reliability cohort 2019 = 0.907
