# Supplementary material for: Medical students’ crisis-induced stress and the association with social support
Source: PLoS One. 2022 Dec 1;17(12):e0278577. doi: 10.1371/journal.pone.0278577 (PMC9714810; doi:10.1371/journal.pone.0278577)
Supplement: Attachment — Submitted filename: Response to Reviewers.docx [file pone.0278577.s002.docx]

Dear Dr. Mughal,

We would like to start with thanking you for your time and efforts regarding our manuscript. We are grateful that you are willing to consider our manuscript for publication and to give us an opportunity to process the feedback in a revised version of our manuscript. Please find below our point-to-point reply and related changes in the manuscript.

We feel that our manuscript has been improved based on the reviewers’ input. We hope that our manuscript can now be accepted for publication.

Yours sincerely,

Andrea M. Woltman, PhD

Associate Professor and Director of Bachelor in Medicine

Erasmus MC University Medical Center Rotterdam, the Netherlands

*Please note that the line numbers we refer to, are the line numbers in the manuscript in which we tracked the changes.*

**Editor**

***1. Please ensure that your manuscript meets PLOS ONE's style requirements, including those for file naming. The PLOS ONE style templates can be found at*** 
[***https://journals.plos.org/plosone/s/file?id=wjVg/PLOSOne_formatting_sample_main_body.pdf***](https://eur01.safelinks.protection.outlook.com/?url=https%3A%2F%2Fjournals.plos.org%2Fplosone%2Fs%2Ffile%3Fid%3DwjVg%2FPLOSOne_formatting_sample_main_body.pdf&data=05%7C01%7Cv.broks%40erasmusmc.nl%7Cfd46e991651346a0cdcc08da3e2cf0d1%7C526638ba6af34b0fa532a1a511f4ac80%7C0%7C0%7C637890660149303173%7CUnknown%7CTWFpbGZsb3d8eyJWIjoiMC4wLjAwMDAiLCJQIjoiV2luMzIiLCJBTiI6Ik1haWwiLCJXVCI6Mn0%3D%7C3000%7C%7C%7C&sdata=qxANlGgjTiZcFUgPn%2F%2F6EI7gn%2BrBep6ZgCGnWtIgmUA%3D&reserved=0) ***and*** 
[***https://journals.plos.org/plosone/s/file?id=ba62/PLOSOne_formatting_sample_title_authors_affiliations.pdf***](https://eur01.safelinks.protection.outlook.com/?url=https%3A%2F%2Fjournals.plos.org%2Fplosone%2Fs%2Ffile%3Fid%3Dba62%2FPLOSOne_formatting_sample_title_authors_affiliations.pdf&data=05%7C01%7Cv.broks%40erasmusmc.nl%7Cfd46e991651346a0cdcc08da3e2cf0d1%7C526638ba6af34b0fa532a1a511f4ac80%7C0%7C0%7C637890660149303173%7CUnknown%7CTWFpbGZsb3d8eyJWIjoiMC4wLjAwMDAiLCJQIjoiV2luMzIiLCJBTiI6Ik1haWwiLCJXVCI6Mn0%3D%7C3000%7C%7C%7C&sdata=v%2FVAg6CNS7rw0unGi7Or5AtvhJQPnlilToeFuxzZ5mM%3D&reserved=0)

We changed the file name of our supplemental file and hope all file names are now in line with PLOS ONE’s style requirements. To the best of our knowledge, the rest of the manuscript was already in line with the style requirements.

***2. Please provide additional details regarding participant consent. In the ethics statement in the Methods and online submission information, please ensure that you have specified what type you obtained (for instance, written or verbal, and if verbal, how it was documented and witnessed). If your study included minors, state whether you obtained consent from parents or guardians. If the need for consent was waived by the ethics committee, please include this information.***

In the methods section, we added that we obtained written informed consent of all participants (underlined part was added to line 129).

*“Students provided written informed consent for the data collected by questionnaires.”*

As already stated in the original manuscript, the study was deemed exempt from further review by the Medical Ethics Committee of Erasmus MC Rotterdam (MEC-2019-0448).

***3. We note that you have indicated that data from this study are available upon request. PLOS only allows data to be available upon request if there are legal or ethical restrictions on sharing data publicly. For more information on unacceptable data access restrictions, please see*** [***http://journals.plos.org/plosone/s/data-availability#loc-unacceptable-data-access-restrictions***](https://eur01.safelinks.protection.outlook.com/?url=http%3A%2F%2Fjournals.plos.org%2Fplosone%2Fs%2Fdata-availability%23loc-unacceptable-data-access-restrictions&data=05%7C01%7Cv.broks%40erasmusmc.nl%7Cfd46e991651346a0cdcc08da3e2cf0d1%7C526638ba6af34b0fa532a1a511f4ac80%7C0%7C0%7C637890660149303173%7CUnknown%7CTWFpbGZsb3d8eyJWIjoiMC4wLjAwMDAiLCJQIjoiV2luMzIiLCJBTiI6Ik1haWwiLCJXVCI6Mn0%3D%7C3000%7C%7C%7C&sdata=AYwd2fp0e13N1Oi%2B8FTjUOokNrdTQGRZGzSnzOCr57g%3D&reserved=0)***.***

***In your revised cover letter, please address the following prompts:***

***a) If there are ethical or legal restrictions on sharing a de-identified data set, please explain them in detail (e.g., data contain potentially sensitive information, data are owned by a third-party organization, etc.) and who has imposed them (e.g., an ethics committee). Please also provide contact information for a data access committee, ethics committee, or other institutional body to which data requests may be sent.***

***b) If there are no restrictions, please upload the minimal anonymized data set necessary to replicate your study findings as either Supporting Information files or to a stable, public repository and provide us with the relevant URLs, DOIs, or accession numbers. For a list of acceptable repositories, please see*** [***http://journals.plos.org/plosone/s/data-availability#loc-recommended-repositories***](https://eur01.safelinks.protection.outlook.com/?url=http%3A%2F%2Fjournals.plos.org%2Fplosone%2Fs%2Fdata-availability%23loc-recommended-repositories&data=05%7C01%7Cv.broks%40erasmusmc.nl%7Cfd46e991651346a0cdcc08da3e2cf0d1%7C526638ba6af34b0fa532a1a511f4ac80%7C0%7C0%7C637890660149303173%7CUnknown%7CTWFpbGZsb3d8eyJWIjoiMC4wLjAwMDAiLCJQIjoiV2luMzIiLCJBTiI6Ik1haWwiLCJXVCI6Mn0%3D%7C3000%7C%7C%7C&sdata=CfNtLdEC2WsgpgqULfzJcVrqI5zllHiXbjV6RwHRSTc%3D&reserved=0)***.***

***We will update your Data Availability statement on your behalf to reflect the information you provide.***

The database contains levels of perceived stress linked to students personal characteristics. Due to the sensitivity of the data we chose to not make these data publicly available.

Data requests may be sent to the institute of Medical Education Research Rotterdam (iMERR). Requests can be sent to associate professor Sílvia Mamede (iMERR): [s.mamede@erasmusmc.nl](mailto:s.mamede@erasmusmc.nl). iMERR can also be contacted via the website: [www.imerr.nl](https://eur01.safelinks.protection.outlook.com/?url=http%3A%2F%2Fwww.imerr.nl%2F&data=05%7C01%7Cv.broks%40erasmusmc.nl%7Ce38c58a0b9174d07c1fb08da4eb36627%7C526638ba6af34b0fa532a1a511f4ac80%7C0%7C0%7C637908829653816683%7CUnknown%7CTWFpbGZsb3d8eyJWIjoiMC4wLjAwMDAiLCJQIjoiV2luMzIiLCJBTiI6Ik1haWwiLCJXVCI6Mn0%3D%7C3000%7C%7C%7C&sdata=vFPFDSSLZB%2BNfr4rkfRo9jBzz6Ct3G0zyBUfSvwHwV0%3D&reserved=0), under the tab “Contact”.

***4. Please include captions for your Supporting Information files at the end of your manuscript, and update any in-text citations to match accordingly. Please see our Supporting Information guidelines for more information:*** [***http://journals.plos.org/plosone/s/supporting-information***](https://eur01.safelinks.protection.outlook.com/?url=http%3A%2F%2Fjournals.plos.org%2Fplosone%2Fs%2Fsupporting-information&data=05%7C01%7Cv.broks%40erasmusmc.nl%7Cfd46e991651346a0cdcc08da3e2cf0d1%7C526638ba6af34b0fa532a1a511f4ac80%7C0%7C0%7C637890660149303173%7CUnknown%7CTWFpbGZsb3d8eyJWIjoiMC4wLjAwMDAiLCJQIjoiV2luMzIiLCJBTiI6Ik1haWwiLCJXVCI6Mn0%3D%7C3000%7C%7C%7C&sdata=qBcm45HZZ9HlUG%2BxaEnk6DG1Lyv4wMkknTvqseXbd6M%3D&reserved=0).

We added the captions of our Supporting Table (S1) at the end of the manuscript.

**Reviewer #1**

***Manuscript #PONE-D-21-39400 examined perceived stress and social support among two different cohorts of Dutch medical students, and tested whether changes in stress and social support were related to the COVID-19 pandemic. This is an interesting sample, and addresses questions that are relevant to stress and coping during the pandemic. Although the paper is written in a very succinct style, many details of the study were clearly articulated. I’ll address my comments below in approximately the order they occur in the manuscript.***

We would like to thank the reviewer for the time taken to read our manuscript and for the kind words about the level of detail, the sample and relevancy of the study.

**Introduction**

***The introduction seemed unnecessarily minimal to me. The authors might want to increase their discussion of social support, especially since they go on to use/describe three different types of social support (perceived informational/emotional and the club membership variable). The club membership variable might best be understood as an indicator of engagement with social networks. This is important because the stress-buffering effects of social support are typically most clearly supported for measures of perceived social support (vs. social network engagement). It would be good to discuss why these measures were used and how they are relevant to medical students during the pandemic.***

We agree with the reviewer that a further elaboration on different forms of social support is of added value in the introduction. We therefore added the following part to the introduction (lines 81-84):

*“Within social support, strong and weak ties can be distinguished, referring to intimate ties with friends or family and non-intimate ties with acquaintances, respectively [23]. According to the literature, both strong and weak ties can positively affect wellbeing and are able to buffer stress [24-26].”*

Including the following new references:

- 23.	Granovetter MS. The strength of weak ties. Am J Sociol. 1973;78(6):1360-80.
- 24.	Sandstrom GM, Dunn EW. Social interactions and well-being: The surprising power of weak ties. Pers Soc Psychol Bull. 2014;40(7):910-22.
- 25.	Thoits PA. Mechanisms linking social ties and support to physical and mental health. J Health Soc Behav. 2011;52(2):145-61.
- 26.	Ferlander S. The importance of different forms of social capital for health. Acta Sociol. 2007;50(2):115-28.

***In addition, the dependent measure was perceived stress, rather than a concept like resilience or other mental health outcomes. It would be useful to more clearly articulate why it is appropriate/useful to use naturally occurring COVID stress as an implicit IV that distinguishes the two cohorts, but also to look at perceived stress as the outcome. The authors hinted at a good reason when they indicated that social support may affect stress appraisal, but it would be better to more fully articulate/justify the approach taken by the study, especially since many of the studies they cite look at mental health outcomes.***

We thank the reviewer for this interesting viewpoint. As we describe in the final paragraph of the introduction, existing studies cannot provide solid evidence of altered stress levels during the COVID-19 outbreak due to lack of required baseline measurements and controls for study performance. We therefore took the opportunity to quantify the COVID-19 induced stress as it is often still a ‘gut feeling’. We agree that there are many ways to operationalize mental health. Stress is the first response to a stressor such as the COVID-19 pandemic, which only leads to other mental health outcomes on the longer term. To underline this, we added the following to the final paragraph of the introduction (see underlined, lines 95-99):

*“The COVID-19 outbreak presented the unique opportunity to assess crisis-induced stress on a large scale for medical students. Stress can be caused by environmental factors such as the COVID-19 outbreak and is linked to both psychological and physical wellbeing in the long-term [7, 39-41]. However, existing studies cannot provide solid evidence of altered stress levels during the COVID-19 outbreak due to lack of required baseline measurements and controls for study performance.”*

Including the following new references:

- 39.	Cohen S, Gianaros PJ, Manuck SB. A stage model of stress and disease. Perspect Psychol Sci. 2016;11(4):456-63.
- 40.	Cohen S, Janicki-Deverts D, Miller GE. Psychological stress and disease. JAMA. 2007;298(14):1685-7.
- 41.	Crosswell AD, Lockwood KG. Best practices for stress measurement: How to measure psychological stress in health research. Health Psychol Open. 2020;7(2):2055102920933072.

***Overall, I found the writing to be clear, but there are some wording oddities that the authors should fix. For example, line 76 says “…social support can prevent that someone appraises…,” which is a very awkward construction.***

The reviewer is right about the awkward construction of this sentence, we adjusted it and hope it now is more pleasant to read (lines 79-80)

*“Social support helps a person to appraise certain events as less stressful.”*

We checked our manuscript for other wording oddities and further improved the writing following the suggestions by reviewer 2.

**Method**

***-I am confused about what is meant by line 122/123. Do the authors mean that they only got aggregate descriptive information on whole cohorts, and that variables like gender and performance are not linked with individual reports of PSS and social support? If so, I’m confused about how those factors were considered statistically. Could you please clarify?
-From the analysis, it looks like pre-and posttest data were paired for both cohorts. How were the responses paired (e.g., student ID number, etc?)?
-Were there missing data/items?***

The reviewer is right that this part of the methods section is unclear. Therefore, we added some extra information to this paragraph to avoid any possible misunderstanding (lines 128-136):

*“Students provided written informed consent for the data collected by questionnaires and to link questionnaire results to relevant data from the student administration. The university student administration provided data regarding gender and study performance up to the final measurement. To link data from different sources, students’ unique identification numbers were used. After linking the data, these student identification numbers were removed from the database. The student administration also provided aggregated data from the complete cohorts, including students that did not complete the questionnaires. Since these data were only reported on an aggregated level, no individual consent was required.”*

Regarding the question about missing data/items, we hope that the following sentence now makes it clear that only complete questionnaires were analyzed (see underlined added words in statistical analysis, lines 186-187):

*“Analyses were only performed on results of students that fully completed both the baseline questionnaire and final questionnaire during Year-1.”*

***-Could you please say more about the shape of the distributions of PSS and social support? I am especially curious about the shape of the distribution of social support since the table notes that it is not normally distributed, but yet it seems to have been analyzed without modification in the regression analyses?***

PSS data demonstrated a normal distribution. Social support was not normally distributed, as the data were left-skewed which is also shown by the median (14/16) in the descriptive table.

In the linear regression analysis, the independent variable is not required to be normally distributed, as long as the residuals are normally distributed. After defining the regression model, we checked all assumptions, including the normal distribution of residuals. The regression model meets all assumptions regarding mean residuals, homoscedasticity, autocorrelation, correlation between predictors and residuals, variability of predictors and multi-collinearity. To provide more information on the analyses, we added the following sentence to the section about statistical analysis (lines 199-201):

*“The regression model met all assumptions and therefore regression analyses were performed without modifications*.”

***-I do not understand the function of the t-tests reported on lines 130-136. Why not just test these together as a 2 x 2 mixed ANOVA that considers time (fall-spring) and cohort (2018-2019)? It doesn’t make sense to separate the t-tests without first conducting the full 2x2.***

The reason we chose the paired t-test in the first place, was simplicity. We do agree with the reviewer that in this case, a two-way mixed ANOVA would be a more elegant solution. However, assumptions underlying the two-way mixed ANOVA regarding homogeneity of (co)variances were not met. Therefore, a combination of the paired t-test and linear regression analysis seems most appropriate: 1) The paired t-test to compare baseline and final measurements *within* cohorts 2) The linear regression model to compare final measurements *between* cohorts in which student characteristics are controlled for (baseline measurement, student gender and study performance).

For now we have chosen to report the paired t-test in combination with the linear regression model, because we think this is the most clear and simple solution. If the editor or reviewer still considers a two-way mixed ANOVA the better option regardless of the violated assumptions, there are two options:

1. We could additionally report the two-way mixed ANOVA results with the side-note that results should be interpreted with caution given the fact that assumptions regarding homogeneity of (co)variances were violated.
2. We could use non-parametric alternatives for the two-way mixed ANOVA, resulting in a combination of the Mann-Whitney-U test (between subjects) and Friedman test (within subjects). We should mention, however, that this still won’t result in a full two-way mixed model as the reviewer suggests. In addition, the results are difficult for readers to interpret.

The authors are open to make further adjustments based on above-mentioned options if the editor or reviewers deem it necessary.

***-Did you consider looking separately at emotional and informational social support? It would be interesting to know whether the results were primarily driven by emotional social support, which likely would have been received from longer term relationships as opposed to new school-based relationships, since social connections were necessarily limited during the pandemic. That’s just speculation, of course, but it would be interesting to know if the results were similar.***

Even though we do think the reviewer makes an interesting point here, the available data in the present study cannot answer this question as the scale was not built to distinguish between these two types of support. We hope the S-1 table provides a more clear idea of the way the emotional-informational scale was set-up based on the MOS social support scale.

We do think the reviewer brings up an interesting viewpoint here, we therefore mention this in the Discussion (lines 336-339):

*“Perhaps, the amount of emotional-informational support did not change but the source of this support did: from school-based relationships to relationships with close family. This could be further explored in future research.”*

***-We are unable to see Table S-1, so unfortunately, I am unclear on the reliability/descriptives of these measures.***

***-As suggested above, it seems like a good idea to justify/contextualize the inclusion of the club measure as an indicator of social support.***

We are sorry to hear that you were unable to see the supplemental file. For both cohorts, the scale measuring emotional-informational support showed an alpha reliability of approximately .9 which indicates a good reliability without redundancy.

We feel the inclusion of club membership now makes more sense due to the added information in the introduction (see above). We now also refer to this addition in the methods section (lines 171-173):

*“Social support was assessed with questions regarding emotional-informational support and club membership to cover both stronger and weaker social ties that are relevant to students in the Netherlands [44].”*

**Results**

***-The logic for the “post-hoc linear regression analysis” described on lines 184-189 was not at all clear to me. What is the purpose of this? Why cut off the continuous variable at the 25th percentile? Were you interested in the interaction between club membership and perceived social support? If so, why? Also if that were the goal, why not center both terms and make an interaction, as you did with the cohort x social support analysis (at least I assume you centered those predictors).***

We regret that the goal of the post-hoc linear regression analysis was not completely clear to the reviewer. The goal of the post-hoc linear regression analysis was mainly to clarify the results of the linear regression model. We wanted to further explore to which degree emotional-informational support and club membership were complementary to each other. We did so by creating four subgroups based on club membership (yes or no) and emotional-informational support (high or low) and comparing stress levels of these subgroups. For emotional-informational support, we decided to create a cut-off on the 25^th^ percentile to distinguish between students having low or high levels of emotional-informational support. The 25^th^ percentile for the cut-off was chosen based on the left-skewness of the distribution of emotional-informational support. This resulted in a group of students that scored lower than the median on emotional-informational support while still maintaining an acceptable group-size.

To clarify this for the reader, we added the underlined sentences to the section about statistical analysis (lines 201-204 & lines 207-208):

*“To clarify the results of the linear regression analysis, post-hoc analyses were performed for both cohorts to assess to which degree emotional-informational support and club membership were complementary to each other in explaining stress levels.”*

*“Emotional-informational support was considered low when students’ scores were in the 25th percentile of the complete sample. This resulted in a reasonable cut-off to select students that scored lower than the median while still maintaining an acceptable group-size.”*

***-There seems to be some sort of suppressor relationship going on with the interaction term. In Model 4 the b value for cohort jumps to 12.44 (from 3.89). Maybe see if this remains the same if the variables are centered (if they weren’t)? I also wonder whether skew in your variables (social support and PSS) might have contributed to this? Did you examine distributions and test for outliers? That might also help clarify the situation. It seems important to think about that change in the magnitude of the effect of cohort on stress in Model 3 (vs 2) and say something about it.***

The reviewer points towards a potential suppressor relationship and proposes to adjust the analysis with centered variables. As stated above, the linear regression model met all assumptions regarding residuals etc. We did not center the social support variable in the original analyses. In response to the reviewers suggestion, we now centered the continuous variables in the linear regression model (PSS-baseline and emotional-informational support) and changed the results in Table 2 accordingly. The magnitude of the shift in b-value between models is now smaller which suggests that a suppressor relationship is not present. The adjusted analysis did neither change the other parts of the model nor the results. The present use of centered variables will avoid possible confusion for the reader and makes the intercept and the cohort effect more meaningful. We thank the reviewer for the suggestion.

For completeness we also added the following sentence in the methods section (line 199):

*“All continuous variables in the regression model were centred for interpretation purposes.”*

**Discussion**

***-Thank you for highlighting the lower response rate in 2019. You might even take that a little further, since the response rate differences are confounded with the quasi-IV of pandemic stress. Were students equally likely to be missing both fall and spring reports in the 2019 cohort? If it data were especially likely to be missing in spring 2020, it would be possible to test whether those who dropped were different in PSS in the Fall (just through a t-test). Often it is the people who struggle the most who fail to continue in a longitudinal study. If that were the case, I think your argument would be that the selection differences underestimate the likely magnitude of the differences between the cohorts. If your most stressed people dropped from the study in Spring 2020, it would raise the mean for Spring 2020 in contrast to Spring 2019… and suggest that the actual difference in stress might be larger. See Cook and Campbell (1979) for a wonderful discussion of these selection effects, and possible selection x maturation effects.***

The authors thank the reviewer for this great and interesting reference. We agree that higher stress levels might be an explanation for the lower response rates. Nevertheless, based on our dataset we are not able to be sure about why participants did refrain from participating in the final measurement. This could either be because they were already so stressed they did not want to be bothered with such a questionnaire, or because they were not stressed and therefore did not think this was a relevant questionnaire for them. The result could therefore either be an underestimation or overestimation of the effect.

To add these different viewpoints to the discussion, we added the following part to the discussion section (lines 375-379):

*“Perhaps, the highly stressed students were less likely to complete the questionnaires because they did not want to be bothered with it. On the other hand, it is also possible that the students who were highly stressed were more likely to complete the questionnaire because of the relevance of the topic to them, opposed to students who were feeling less stressed. This may have led to an underestimation or overestimation of the effect.”*

**Reviewer #2**

***The manuscript regarding university students and Covid was interesting and pertinent to the journal. The only issues were some of the tense used throughout was not correct (see below) with some punctuation issues. This reviewer commends the authors in their research.***

We would like to thank the reviewer for the time taken to read our manuscript and for the kind words about the sample and relevancy of the study.

***Line 46, eliminate the dash after anxiety and depression.
Line, 53, eliminate the "-", maybe use a comma in place of them?
Line 55-57, use had instead of has, since the study is in the past. Same comment for the next two sentences, use was instead of is.
Line 64, change studies show to studies showed or use studies reported, but add the studies in this sentence.
Line 65, change review reports to reported.
Line 66, same comment as above, use reported.
Line 76, "Firstly, because..." not a complete sentence, please reword.
Line 77, "Secondly, because.."... is not a complete sentence, please reword.
Line 79, neighbours or neighbors?
Line 80, change shows to showed or reported.
Line 82, change indicates to indicated.
Line 83, change is to was
Line 92, check the punctuation, the dashes are not the same, maybe use commas?
Line 96, change is to was, since you did complete the student.
Line 106, make 6o, 60, use the actual zero, not the letter "o"
Line 133, change are to were.
Line 134, change is to was.
Line 141, change are to were
Line 142, change gender is to "gender was," change "performance is" to "performance was".
Line 150, change questionnaire is to "was".*** *🡪 because this applies to the present we did not change this*
***Line 165, change are to was.
Line 166, change is to was.
Line 202, change is to was.
Line 221, confusing that "a" reference category is male, but above is female?*** *🡪 we hope the adjustments made to the table create more clarity* ***Line 232, change is to was.
Line 238, add a comma after 2018.
Line 241, change indicate to indicated.
Line 245, delete the dash, add a comma.
Line 269, change are to was.
Line 270, change do to did.
Line 271, change do to did.
Line 275, change demonstrates to demonstrated
Line 280, change indicate to indicated, is to was.
Line 302, change report to reported.
Lines, 323 and 324, delete the dashes and add commas.***

The authors are grateful for the reviewer’s detailed feedback regarding tense and punctuation. We have made adjustments to the manuscript according to the suggestions of the reviewer in order to make it more appropriate. We have added some additional responses to abovementioned points, where indicated (see above, indicated with “🡪”).

Moreover, we critically revised the manuscript and made some extra adjustments to improve readability.
