# Supplementary material for: Medical students’ crisis-induced stress and the association with social support
Source: PLoS One. 2022 Dec 1;17(12):e0278577. doi: 10.1371/journal.pone.0278577 (PMC9714810; doi:10.1371/journal.pone.0278577)
Supplement: Attachment — Submitted filename: Response to reviewers.docx [file pone.0278577.s003.docx]

**Response to reviewers**

Reviewer #1: I have carefully reevaluated manuscript PONE-D-21-39400_R1, and appreciate the authors’ thorough attention to many of the issues raised in the previous reviews. I still think this manuscript is interesting and makes a good contribution to he literature. There are some remaining questions/concerns the authors and editor should probably consider as well. My most pressing concerns relate to the data analysis.

We want to thank Reviewer #1 for the time and critical review. We are pleased that the reviewer appreciates our previous reply to most issues. We addressed the remaining concerns or ambiguities below, and made changes in the manuscript accordingly. We think that these changes have further improved the manuscript.

Please note that the line numbers we refer to in this response, are based on the revised manuscript in which we tracked the changes.

There are still some issues with the wording and use of tense throughout the manuscript. Perhaps a copy editor can assist with these details? For example, the sentence on line 33/34 in the abstract is unclear. Maybe instead say something like “Baseline stress measurements for the 2018 and 2019 cohorts did not differ significantly.” I also had to re-read the sentences on lines 97-98 and 185-188 to make sense of them. Line 242 should say memberS of a club… The use of “beside each other” in line 254/255 is awkward. Please review tense throughout—I noticed especially on p. 18 (lines 309-316).

We thank the reviewer for these observations. As a result, we critically revised the manuscript and asked a native English speaker the check the manuscript. We made adjustments accordingly:

- Line 33-35 (reviewer referred to as line 33/34):
  - Old: “Stress levels did not differ statistically significant between both pre-COVID-19 measurements of cohort 2018 and baseline cohort 2019.”
  - New: “In cohort 2018 (pre-COVID-19), students’ perceived stress levels did not differ significantly between the baseline and final measurements. Additionally, baseline measurements of the two cohorts (2018 and 2019) were not found to be significantly different.”
- Line 110-112 (reviewer referred to as line 97/98):
  - Old: “Even though medical schools are unable to resolve the present COVID-19 crisis or any future crisis, they might be able to help limit the negative consequences on student wellbeing, which is already relatively low.”
  - New: “Even though medical schools are unable to resolve the present COVID-19 crisis or any future crisis, they might be able to limit the negative consequences on student wellbeing.”
- Line 200-206 (reviewer referred to as line 185-188):
  - Old: “Then, to study the impact of COVID-19 on perceived stress, paired t-tests were performed on baseline stress levels and related final measurement stress levels of both cohort 2018 and cohort 2019. In addition, stress levels during the final measurement of cohort 2018 (pre-COVID-19) and cohort 2019 (COVID-19) were compared in a linear regression model while controlling for baseline stress level, gender and study performance.”
  - New: “Changes in stress levels during the COVID-19 outbreak were assessed using multiple methods. Firstly, paired t-tests were performed in both cohorts to assess whether stress levels within the cohorts changed between their baseline and final measurement. Secondly, final stress level measurements of cohort 2018 (pre-COVID-19) and cohort 2019 (COVID-19) were compared in a linear regression model while controlling for baseline stress level, gender, and study performance.”
- Line 262-264 (reviewer referred to as line 242):
  - Old: “Also, students who were member of a club had significant lower stress levels compared to their fellow students who were not member of a club (B_club member_=-1.636, 95%-CI: -3.197 – -0.077, p<.05, Table 2 – Model 2).”
  - New: “Also, students who were club members had significant lower stress levels compared to their fellow students who were not (B_club member_=-1.636, 95%-CI: -3.197 – -0.077, p<.05, Table 2 – Model 2).”
- Line 276 -278 (reviewer referred to as line 254/255):
  - Old: “The results indicated that for cohort 2019, emotional-informational support and club membership were complementary to each other in relation to stress levels since the effects exist beside each other.”
  - New: “These results suggest that both emotional-informational support and club membership were associated with lower stress levels for students in cohort 2019.”
- Line 336-344 (reviewer referred to as line 309-316):
  - Old: “In line with the stress-buffering model [10, 11], we found that only during the COVID-19 outbreak, higher levels of social support were associated with lower stress levels for medical students. This shows that also during a crisis in which social contacts are strained by social distancing measures, social support is associated with lower stress levels. This mechanism is also corroborated by a recent study in which medical students report that their own most effective strategies to lower the negative impact of stress include de-stress through friends and family [49]. Studies reported that in times of the COVID-19 outbreak, social support is positively associated to wellbeing in adolescents [30], college students[31], and the general population [28].”
  - New: “In line with the stress-buffering model [10, 11], we found that only during the COVID-19 outbreak, in which social contacts were strained by social distancing measures, higher levels of social support were associated with lower stress levels for medical students. This mechanism has also been confirmed by a recent study in which medical students reported that their own most effective strategies to lower the negative impact of stress included de-stress through friends and family [50]. In line, in times of the COVID-19 outbreak, social support was found to be positively associated with wellbeing in adolescents [31], college students[32], and the general population [29].”

Additionally, we made several adjustments throughout the manuscript to improve grammar, wording, and tense based on both the feedback of the native speaker and our critical re-reading.

I believe that clarity would be greatly improved if the authors used a term like “perceived social support” instead of "emotional-informational" support. If the authors want to keep the E-I support label they should explain the construct and use the term “emotional-informational” support in the introduction. Right now the first time the readers see that term is in the method section, and so it adds bulk and confusion throughout the last half of the paper. My sense is that the authors are using the MOS subscale as a measure of perceived social support, and so that label might work well. It looks like it is mainly an emotional social support measure. Only the last question appears to deal with informational support, and even that item seems to focus more on whether the person feels like they have someone available for informational support, rather than on the actual receipt of informational social support.

Likewise, although the discussion of social support is much improved in the introduction, there is still not a clear link to the club variable in the method section. School clubs could easily be added as an example of a setting that might be more likely to foster weak social ties (around line 82), so the reader was better prepared to see the club variable as a proxy social support variable.

We are pleased with the reviewer’s recognition of the improved introduction. We also appreciate the reviewer’s concern regarding the clarity of social support. We understand that the reviewer suggests that we either explain emotional-informational support in the introduction or change the term to perceived or emotional social support.

We used questions from the emotional-informational scale, consisting of items on both emotional and informational items:

- Emotional: concerning having someone that listens to you and having someone to share worries or fears with
- Informational: concerning having someone that offers advice or suggestions

Since the scale we used includes emotional and informational support, we chose to introduce these terms in the introduction, as the reviewer suggested. We agree with the reviewer that this improves clarity. Furthermore, the reviewer suggests to add a clear link in the introduction to the use of the club membership variable in the methods section. Therefore, we also added a part in the introduction about club membership.

We added a part in the introduction, line 85-92:

“Strong and weak ties can be distinguished in social support systems, such as intimate ties with friends or family and non-intimate ties with acquaintances, respectively [23]. Intimate ties (i.e., strong ties) can offer emotional-informational support, composed of an emotional and informational component. The emotional component consists of care, love, and empathy (e.g., providing a listening ear) [24]. The informational component consists of guidance to offer a solution for a problem (e.g., providing advice) [24]. Non-intimate ties (i.e., weak ties), such as ties with fellow members of a sports- or hobby club, are less personal [23, 25], but can offer social companionship involving time spent with others in activities of recreational nature [24].”

We also added a part in the research question (line 117-118, see underlined):

“Did social support, broken down into emotional-informational support and club membership, moderate the effect of the COVID-19 outbreak on the perceived stress of medical students?”

Either the text or Table 1 should include standard deviations together with means.

We agree with the reviewer that a measure of variance is necessary. Therefore, we provided standard errors in the legends of Figure 1 in the original manuscript. For completeness, we now also added the standard deviation (sd) in Table 1 for the normally distributed variables (the PSS).

Given that the emotional-support variable was non-normally distributed, it would not make sense to report means and standard deviations as a measure of variance. Therefore, we stick to reporting the median and range.

I still did not find an analysis of attrition. It would be very useful to know if the pretest stress level differed between those people who remained in the study and those who dropped out. This would help empirically address the question of whether the people who completed the spring measure (in both cohorts) were more or less stressed than those who remained in the study. This could be done with a 2 x 2 between ANOVA (first IV would be whether the participants completed the spring assessment, second would be 2018/2019 cohort) with baseline stress as the DV. This would indicate whether those who dropped out were more/less stressed in the fall, and also whether that pattern differed by cohort. If for some reason that isn’t possible, you could instead use two between-subjects t-tests to test each cohort separately.

As the reviewer states, it would be useful to know whether the baseline stress level differed between those people who remained in the study and those who dropped out. This information indicates whether those who dropped out were more or less stressed in the fall, and also whether that pattern differed by cohort. We executed the suggested t-test for both cohorts and found no significant differences in baseline stress levels for students who did vs. did not complete the spring measurement (cohort 2018: t=-0.220, df=172.87, p=.826; cohort 2019: t=1.229, df=198.15, p=.221).

We want to point out that we still do not know how stress levels might have changed during the COVID-19 outbreak of the group who did not complete the final questionnaire. Therefore, we thought the added part in the previous revision round was sufficient. However, as the readers of the manuscript might have the same question as the reviewer did, we added the following to the discussion (line 394-399):

“For both cohorts, students who only completed the baseline measurement did not have significantly different stress levels compared to students who completed both measurements (cohort 2018: t=-0.220, df=172.87, p=.826; cohort 2019: t=1.229, df=198.15, p=.221). Despite these results and the fact that we controlled for student characteristics in the analysis, a selection bias may still be present due to the lower response rate.”

I appreciate the additional details about the analyses and tests of assumptions. That said, extreme outliers can still have a dramatic effect on regression results. Did the authors look at the extent to which those very low in SS may be driving the effects? I especially wonder about this because of the choice to look at the lower/upper quartile in the follow-up analyses. I find it odd not to specifically consider the possible effects of outliers, especially with a predictor with such strong negative skew. It is a little hard for me to understand the overall effects since no means/standard deviations were provided for the social support variable. I wonder if the slope of the relationship between social support and stress would remain stat sig for the 2019 cohort without the couple data points with very low social support. Something like a log transformation of the social support variable (reflected) would help determine whether the extreme scores were driving the results. Tabachnick and Fiddel’s Multivariate Stats book has a great chapter on the effects of distributions/outliers methods for handling them.

We thank the reviewer for the appreciation of the additional details about the tests of assumptions. The reviewer raised a relevant point regarding the potential effects of extreme outliers on regression results. We understand that the reviewer is worried whether outliers on the emotional-informational support variable drive the regression results. We tried the suggested solution offered by the reviewer regarding the log transformation. However, this was not effective as the log transformed social support variable still did not show a normal distribution. We have further investigated how we could study the potential impact of outliers on a regression analysis. Cook’s distance is one of the measures that is described in the multivariate statistics book by Tabachnik and Fidell that the reviewer referred to. We found that Cook’s distance is the most fitting way to identify observations that had most significant impact on the regression results. ^[1]^

We took the following steps:

- **Step 1:** We computed Cook’s distance for each observation based on linear regression model 3 (the model including the interaction effects). We computed Cook’s distance for each observation, representing the overall influence of an observation on the model.
- **Step 2:** The literature states that Cook’s distance values higher than 1 cause concern. We identified no values of Cook’s distance larger than 1. The largest value for Cook’s distance was 0.065.
- **Step 3:** Even though no alarm bells went off based on step 2, we also decided to look at the most influential values based on Cook’s distance by marking values larger than 4/n (where n is the number of observations). This value is a rule of thumb described in the literature to identify the most influential observations.^[2]^ Based on this method, we found 19 observations having the largest effect on the regression results. We thought the reviewer might like to see for each cohort combined with the emotional-informational support score which cases were most influential:

| **Cohort** | **Emotional-informational support score** | **# cases** |
| --- | --- | --- |
| 2018 | 6 | 1 |
| 2018 | 10 | 1 |
| 2018 | 12 | 1 |
| 2018 | 14 | 1 |
| 2018 | 16 | 2 |
| 2019 | 8 | 1 |
| 2019 | 10 | 4 |
| 2019 | 12 | 2 |
| 2019 | 14 | 1 |
| 2019 | 16 | 5 |
| **Total** |  | **19** |

Based on these results, we see that of the 19 influential cases, most cases entail students with a relatively high emotional-informational support score in 2019.

- **Step 4:** To explore to which degree these influential cases impacted the regression results, we re-ran the linear regression analysis while excluding above-identified cases with the following results:

|  | **Model 3**  **Most influential cases excluded**  **(n=277, 18 observations excluded)** | | |
| --- | --- | --- | --- |
|  | **B** |  | **sig.** |
| ***Intercept*** | *26.571* |  | *-* |
|  |  |  |  |
| **PSS - baseline^a^** | 0.579 |  | *** |
| **Female** (ref: male) | 2.650 |  | ** |
| **All credits obtained** (ref: not all credits) | -3.309 |  | *** |
|  |  |  |  |
| **Cohort 2019 - COVID-19** (ref: 2018) | 5.477 |  | *** |
|  |  |  |  |
| **Emotional-informational (E-I) support^b^** | -0.196 |  |  |
| **Club member: yes** (ref: no) | -0.782 |  |  |
|  |  |  |  |
| **Cohort (2019) * E-I support** | -0.634 |  | ** |
| **Cohort (2019) * Club member (yes)** | -3.327 |  | * |
|  |  |  |  |

This analysis regarding Cook’s distance (step 1 to 4) show:

- The students with lower emotional-informational support scores were not influential on the linear regression outcomes.
- The regression coefficients reported in the manuscript do not shift dramatically when excluding the influential cases identified by Cook’s distance.

A statistical basis is lacking to exclude any observations since the largest value for Cook’s distance was far below 1 and the regression equation was not dramatically affected by specific observations. We added the following to the methods section to provide the reader also with the information that we looked at Cook’s distance to assess the impact of potential influential observations (line 212):

“The regression model met all assumptions, and Cook’s distance revealed no observations that had a large impact on the regression equation [46].”

Added reference:

- [46] Cook RD, Weisberg S. Residuals and influence in regression: New York: Chapman and Hall; 1982.

I am a bit confused about the concern re: homogeneity of covariance (for using a 2 x 2 ANOVA instead of separate t-tests (line 226). My understanding is that the assumption of sphericity/homogenity of covariance is only relevant when the within subjects variable has more than two levels. Could the authors provide a bit more detail or a reference on their logic here? Maybe I’m misunderstanding or forgetting something?

We are sorry that the reviewer feels confused about our response regarding the assumptions of homogeneity of (co)variance. One assumption of the two-way mixed ANOVA is that variances between groups are equal. This assumption is violated for the final measurement. The variance of final stress levels between cohort 2018 and 2019 is not equal as is confirmed by Levene’s test (F(1,293)=6.28, p<.05). Therefore, a two-way mixed ANOVA would be less optimal to report compared to the analyses currently reported in the manuscript.

Although not relevant as stated above, we would like to show the reviewer that the two-way mixed ANOVA would not have impacted the results and conclusions drawn in the manuscript. The results of the two-way mixed ANOVA show the following:

- A significant main effect for time (i.e. the measurement moments) on PSS scores (F(1,293)=35.434, p<.001).
  - For *cohort 2018*, the baseline and final measurements did not differ statistically significantly
  - For *cohort 2019* the baseline and final measurements did differ statistically significantly (F(1,98)=36.9, p<.001).
- A significant main effect of cohort on PSS scores (F(1, 293) = 9.297, p<.01)
  - During the *baseline measurement*, PSS scores did not differ statistically significantly between cohorts.
  - During the *final measurement*, the PSS score was significantly higher for cohort 2019 compared to cohort 2018 (F(1,293)= 20.2, p<.001).
- A significant interaction effect of Cohort*Time (F(1,293)=20.638, p<.001).

The post-hoc tests described in the text (considering both club membership and social support) would usually be conducted as a follow-up to an interaction. Did the authors test for a 3-way interaction between cohort, club membership, and social support? That is implicitly what is being compared through those quartile examinations, so it seems a bit strange to test this combination of variables without first testing for the 3-way interaction.

We regret that the post-hoc test’s goal was not clear to the reviewer. It was not our intention to suggest a three-way-interaction analysis. The sole purpose of the post-hoc analysis is to illustrate what the results reported in Table 2 – Model 3 look like. We meant to illustrate the additive effects of club membership and social support. To avoid any misunderstanding, we made the following adjustments:

- We are no longer calling it a post-hoc analysis as it is an illustration of the results reported in Table 2 – Model 3.
- We use the word “illustrate” instead of “assess”.

Under the Methods section (line 213-218):

- Old: “To clarify the results of the linear regression analysis, post-hoc analyses were performed for both cohorts to assess to which degree emotional-informational support and club membership were complementary to each other in explaining stress levels. To this end, groups based on social support were formed by crossing the presence of club membership (yes/no) with emotional-informational support (high/low).”
- New: “Finally, to illustrate the results of the linear regression analysis, four subgroups were formed based on the presence of club membership (yes/no) and emotional-informational support (high/low).”

Under the Results section (line 280-281):

- Old: “Accordingly, post-hoc analysis showed that for cohort 2019, emotional-informational support and club membership were complementary to each other in relation to stress levels. Students with only one of the two types of social support –club membership *or* high emotional-informational support - showed significantly higher stress levels compared to students with both types of social support (Fig 3).”
- New: “Fig 3 illustrates that cohort 2019 students with only one of the two types of social support –club membership *or* high emotional-informational support - showed significantly higher stress levels compared to students with both types of social support. For cohort 2018 students, differences in stress levels based on social support were not present.”

Please take care to use the words increase/decrease only when discussing the differences over time in the PSS variable. For example, line 249 discusses E-I social support increasing, but since that was only a cross-sectional measure, so describing correlations as indicating “increases” in social support is misleading. The authors are describing the fact that those who reported MORE social support showed lower stress (decreased stress). I’d also recommend removing decrease from line 322 to avoid any implication that that was a longitudinal comparison.

We thank the reviewer to address the use of words increase/decrease. We agree and therefore made the adjustments accordingly:

- Line 270-271
  - Old: “For cohort 2019, a significant decrease in stress levels was visible as emotional-informational support increased.”
  - New: “Only for cohort 2019, stress levels were significantly lower for students with higher levels of emotional-informational support (Fig 2A).”
- Line 348-358
  - Old: “Even though the present study did not reveal lower levels of emotional-information support during the COVID-19 outbreak, the number of students who reported being member of a club did decrease from 68% to 56%. This decrease is possibly the result of measures taken to limit the spread of the coronavirus, making it impossible for clubs to gather with their members. A potential explanation why emotional-informational support did not decrease is that students moved back home to their parents’ house where emotional-informational support was still available to them since close family is a source of social support for Dutch students [44].”
  - New: “Although students did not report lower levels of emotional-informational support during the COVID-19 outbreak, the number of students who reported being clubmembers was lower during the COVID-19 outbreak than before (56% vs. 68%). This lower percentage is possibly the consequence of measures taken to limit the spread of the coronavirus, making it impossible for clubs to gather with their members. Emotional-informational support was not lower during the COVID-19 outbreak. A potential explanation is that students moved back home to their parent’s house where emotional-informational support was still available since close family is a source of social support for Dutch students [45].”

Please also be careful about using causal language about the role of COVID-19. For example, line 303 discusses the idea that the COVID-19 outbreak ELEVATED stress levels… but yet all we know is that changes in stress were greater in the 2019 cohort than in the 2018 cohort. Those cohorts include different people, less contact with faculty, and many other differences in addition to the pandemic. It would be hard to imagine that the pandemic wasn’t an important factor, but we can’t draw causal conclusions from this study. Also eliminate the term “COVID-19 induced stress” in line 308.

We thank the reviewer for the critical remark about causal language. The reviewer points out a good nuance. We, therefore, made the following adjustments:

- Line 329-331:
  - Old: “The reason why the COVID-19 outbreak elevated stress levels goes beyond the scope of the present study, but one might speculate about a mix of academic uncertainty [8, 17], online education [20], blurred study-home boundaries and social isolation [48].”
  - New: “The reason why stress levels were elevated during the COVID-19 outbreak goes beyond the scope of the present study, but one might speculate about a mix of academic uncertainty [8, 17], online education [20], blurred study-home boundaries, and social isolation [49].
- Line 334-335:
  - Old: “Such baseline measurements could also serve future research on the long-term effects of this COVID-19 induced stress.”
  - New: “Such baseline measurements could also serve future research on the long-term effects of the COVID-19 outbreak.”

To add an extra nuance regarding causality that is unknown, we added the following to the limitations in the discussion (lines 404-407):

“Another limitation of the present study is that we investigated the levels of perceived stress, but not the cause of perceived stress. Higher stress levels during the COVID-19 outbreak could have been caused by either direct effects of the pandemic itself, or indirect effects due to changes in the student’s environment (e.g., living situation, less contact with the faculty).”

Reviewer #2: Well done article. These authors addressed all of the points that this reviewer had made, plus the additions of several paragraphs did make the manuscript stronger.

We are happy to read that all the reviewer’s points were addressed. We thank the reviewer for the kind words.

1. Cook, R. D., & Weisberg, S. (1982). Residuals and influence in regression. New York: Chapman and Hall.
2. Van der Meer, T., Te Grotenhuis, M., & Pelzer, B. (2010). Influential cases in multilevel modeling: A methodological comment. American Sociological Review, 75(1), 173-178.
